# Feature Collection in Peptide Therapeutics: Current Applications and Future Directions

**DOI:** 10.3390/biomedicines12122919

**Published:** 2024-12-23

**Authors:** Bernard Lebleu

**Affiliations:** Laboratory of Host Pathogens and Immunity, Université Montpellier, 34095 Montpellier cedex 5, France; marinaetbernard@lebleu.net

The use of peptides in medicine began long ago with peptidic hormones. Developments in structural biology and molecular biology have led to significant improvements in our knowledge of protein structures, their physiological roles and their involvement in diseases. The advent of recombinant DNA technology has also led to advances in peptide chemistry and the enhanced application of tools for the development of drugs has enabled the rapid expansion of the field.

**Peptide-based medicines** [1,2] now represent an important segment of the pharmaceutical market, with applications in the treatment of many human diseases. Several have become » blockbusters » like, for example, Sanofi Lantus^TM^ recombinant insulin, which is now employed in the treatment of diabetes. More recently, Eli Lilly and Novo Nordisk GLP-1 (glucagon-like peptide-1) receptor agonists, initially developed for type-2 diabetes, have been approved for the treatment of obesity in patients at risk of cardiovascular disease; great commercial success has been achieved in this regard. More than a hundred peptides have been approved by US and European regulatory agencies, and several hundred are currently undergoing pre-clinical or clinical trials. It is estimated that the global market is now approximately USD fifty billons, with an expected annual growth rate of 9–10%.

Peptides can be isolated from natural sources and benefit from the ongoing exploration of microbial, animal and plant species in terrestrial and marine environments, including extreme environments. For example, a screening program performed by Sandoz Ltd. (Basel, Switzerland) in 1971 led to the isolation of cyclosporin (which became a leading immunosuppressive drug) from the *Tolypocladium inflatum* fungus in a Norwegian soil sample. Peptides can also be produced at a reasonable cost via chemical synthesis using solid- or liquid-phase methods, as well as using recombinant technologies.

This increased interest in peptide-based medicines (typically fewer than 50 amino acids) has been driven by their higher efficacy, specificity and safety compared to low-molecular-weight drugs. Crucially, peptides can reach **undruggable targets** [3] that are difficult or impossible to attain with conventional low-molecular-weight drugs.

Numerous clinical applications have been considered in consideration of the highly diverse biological role of peptides as cell structural components, enzyme inhibitors, hormones, neurotransmitters, cell surface receptors or antimicrobials.

The major **limitations of peptide-based drugs** are their susceptibility to protease degradation, poor oral bioavailability, non-specific interactions with plasma proteins and low membrane permeability, which remains a critical issue when addressing intracellular targets.

These limitations of peptide-based drugs will hopefully benefit from current advances in medicinal chemistry and SAR (structure–activity relationship) studies. The chemical modification of backbones or side chains can be performed to increase the metabolic stability or affinity of these drugs for their targets. This includes the site-specific insertion of unnatural amino acids, cyclization or conformational constraints (stapled peptides for example), as well as conjugation with various chemical entities such as polyethylene glycol or fatty acids.

It would be impossible to comprehensively address the many potential **targets of peptide-based drugs and peptidic drug delivery strategies** in this Editorial, so we will limit ourselves to a few examples.

Large-scale screenings using cell biology-based technologies or computational strategies have enabled us to unravel the mechanisms of protein interactomes. Most biological processes are now considered to be regulated by complex **protein–protein interactions** (PPI) [4], with approximately 600,000 interactions occurring in humans. Most PPIs are considered to be undruggable since the interfaces between proteins generally exhibit large surfaces (compared to receptor–ligand interactions), which are often formed between discontinuous sets of amino acids and thus lack well-defined binding sites for low-molecular-mass drugs. While still in its infancy, the field provides many potential targets for the design and development of peptide drugs.

**Antimicrobial peptides** (AMPs) [5] constitute another class of drugs with potentially important applications, particularly considering the threat represented by microbial infections for which drugs are either not available or inefficient due to the increasing number of antibiotic multi-resistant strains. Interestingly, AMPs are produced by nearly all living species as a first line of defense against pathogens, with gramicidin serving as an example of clinical application. Delineating their mode of action (particularly their interactions with the membranes of pathogens), increasing their production and potential, decreasing their side effects and enlarging their spectrum of action may lead to interesting developments and enhance the use of AMPs in clinics.

The limitations of peptide-based strategies could be addressed via their association or encapsulation with **delivery vectors** such as nanoparticles or cell-penetrating peptides. Naturally occurring or synthetic **cell-penetrating peptides** (CPPs) [6] have received considerable attention in recent decades due to their ability to deliver a macromolecular payload (peptide or nucleic acid) across biological membranes. Direct translocation and endocytosis through non-receptor-dependent mechanisms have both been proposed. A better understanding of this topic will hopefully lead to improvements in, for example, the endosomal release of transported drugs. The lack of cell specificity in naturally occurring CPPs is another limitation. Interesting developments include the modification of CPPs, leading to the delivery of their payload in a specific environment through, for instance, tumor-cell-associated enzymes. Other strategies involve the selection of cell-specific CPPs from peptide phage display libraries.

The **development of peptides with optimal pharmacological properties** relies upon the use of a lead version. Ala scans are often used to identify amino acids that can be modified or replaced without affecting biological properties. Improving stability, safety or delivery is often a delicate operation, since minor alterations in the peptide sequence can have profound effects on efficacy or specificity. The field has benefited from technological improvements such as rational designs, phage display, mRNA display or computational methods. In particular, **Artificial Intelligence** (AI) [7] has led to remarkable improvements in peptide-based drug design and therapeutics for the assessment of pharmacodynamic properties or safety.

In this regard, the 2024 Nobel prize in Chemistry has been awarded in part to Demis Hassabis and John M. Jumper (Google DeepMind Labs, London, UK) for the development of the AlphaFold2 AI-based tool, which can be used to **predict three-dimensional protein structures** with much greater accuracy than previous programs; this is a problem that chemists and biologists have tried unsuccessfully to tackle for several decades. This will undoubtedly accelerate the design of more specific or effective protein-targeting drugs, including peptide-based ones.
